# Ecological Niche Modeling of *Cryptococcus gattii* in British Columbia, Canada

**DOI:** 10.1289/ehp.0901448

**Published:** 2009-12-17

**Authors:** Sunny Mak, Brian Klinkenberg, Karen Bartlett, Murray Fyfe

**Affiliations:** 1 Epidemiology Services, British Columbia Centre for Disease Control, Vancouver, British Columbia, Canada; 2 Department of Geography and; 3 School of Environmental Health, University of British Columbia, Vancouver, British Columbia, Canada; 4 Office of the Chief Medical Health Officer, Vancouver Island Health Authority, Victoria, British Columbia, Canada

**Keywords:** British Columbia, cryptococcal disease, cryptococcosis, *Cryptococcus gattii*, ecological niche modeling, fungal disease, GARP, Genetic Algorithm for Rule-set Prediction, geographic information systems, GIS

## Abstract

**Background:**

*Cryptococcus gattii* emerged on Vancouver Island, British Columbia (BC), Canada, in 1999, causing human and animal illness. Environmental sampling for *C. gattii* in southwestern BC has isolated the fungal organism from native vegetation, soil, air, and water.

**Objectives:**

Our aim was to help public health officials in BC delineate where *C. gattii* is currently established and forecast areas that could support *C. gattii* in the future. We also examined the utility of ecological niche modeling (ENM) based on human and animal *C. gattii* disease surveillance data.

**Methods:**

We performed ENM using the Genetic Algorithm for Rule-set Prediction (GARP) to predict the optimal and potential ecological niche areas of *C. gattii* in BC. Human and animal surveillance and environmental sampling data were used to build and test the models based on 15 predictor environmental data layers.

**Results:**

ENM provided very accurate predictions (> 98% accuracy, *p*-value < 0.001) for *C. gattii* in BC. The models identified optimal *C. gattii* ecological niche areas along the central and south eastern coast of Vancouver Island and within the Vancouver Lower Mainland. Elevation, biogeoclimatic zone, and January temperature were good predictors for identifying the ecological niche of *C. gattii* in BC.

**Conclusions:**

The use of human and animal case data for ENM proved useful and effective in identifying the ecological niche of *C. gattii* in BC. These results are shared with public health to increase public and physician awareness of cryptococcal disease in regions at risk of environmental colonization of *C. gattii*.

*Cryptococcus gattii* appeared on Vancouver Island, British Columbia (BC), Canada, in 1999, causing human and animal illness (Duncan et al. 2005; Kidd et al. 2004; MacDougall et al. 2007; Stephen et al. 2002). Cryptococcal disease is initiated by inhalation and infection by microscopic-size fungal propagules (yeast cells or spores ~ 1–2 μ size) through environmental exposure. Cryptococcosis is a potentially fatal infection of the lungs and central nervous system. By the end of 2008, > 240 documented human cases and 360 animal cases had been reported in BC. These cases represent the highest incidence rate of *C. gattii* infection and the only documented multispecies outbreak of cryptococcosis in the world (Bartlett et al. 2007; MacDougall et al. 2007; Stephen et al. 2002).

*C. gattii* has been isolated in BC from native vegetation, soil, air, and water on the south and central eastern coast of Vancouver Island and, more recently, on the Gulf Islands, the Lower Mainland, and in the states of Washington and Oregon (Bartlett et al. 2007; Byrnes et al. 2009; Datta et al. 2009; Kidd et al. 2004, 2007a, 2007b; MacDougall et al. 2007; Upton et al. 2007). Before its discovery in the temperate rainforest region of northwestern North America, *C. gattii* infections and environmental exposures were described in tropical and subtropical regions of the world such as Australia, Africa, India, Italy, Papua New Guinea, South America, and southern California (Casadevall and Perfect 1998; Ellis and Pfeiffer 1990; Kwon-Chung and Bennett 1984; Sorrell 2001).

The epidemiology, genetic characterization, geographic distribution, and environmental sampling for *C. gattii* in BC have been well described elsewhere (Bartlett et al. 2007; Datta et al. 2009; Duncan et al. 2005; Fraser et al. 2005). The purpose of our study was to help public health officials in BC delineate where *C. gattii* is currently established and forecast areas that could support it in the future. Because it is not feasible to sample every location in the province for the presence or absence of *C. gattii*, we used ecological niche modeling (ENM) to predict the spatial distribution of *C. gattii* by relating the environmental characteristics of field observations to predictor variables. We used the Genetic Algorithm for Rule-set Prediction (GARP) to model the ecological niche of *C. gattii* in BC. We also examined the utility of ENM based on human and animal *C. gattii* disease surveillance data.

## Environmental characterization of C. gattii

To date, descriptions of the ecological niche of *C. gattii* have varied with the region under study. General characterizations from studies in Australia (Ellis and Pfeiffer 1990) and Colombia (Granados and Castaneda 2006) describe very different environmental conditions favorable to colonization and aerosolization. In Australia, Ellis and Pfeiffer (1990) suggested the sudden appearance and dispersal of *C. gattii* coincided with the flowering of *Eucalyptus camaldulensis* during late spring, whereas Granados and Castaneda (2006) reported an increase in the rate of positive *C. gattii* samples during the wet and humid months of April and May in Colombia, and identified an association between rainy climatic conditions and increased occurrence of *C. gattii* serotype B in trees in the temperate climate of Bogotá, specifically, high precipitation and humidity, low sunshine hours, and less extreme temperatures during the rainy months.

In BC, Kidd et al. (2007b) did not observe a clear seasonal pattern for *C. gattii* positivity. However, air samples taken during the summer months, which are associated with warm, dry conditions, and low relative humidity, were more likely positive. Kidd et al. (2007b) also observed *C. gattii* from soils within the pH range of 4.33–7.48, where its presence was associated with lower moisture content and lower organic carbon content. Furthermore, *C. gattii* was isolated from both freshwater and seawater: survival assays in the laboratory revealed a high survival rate of *C. gattii* in seawater at 4°C—broadly representing the natural seawater conditions of BC. Finally, *C. gattii* was also isolated from inanimate, nonorganic objects such as vehicle tires, wheel wells, and shoe bottoms that had recently traveled through the endemic *C. gattii* areas on Vancouver Island (Kidd et al. 2007b).

## The ecological niche

The concept of the ecological niche was proposed by Grinnell (1917), who described the ecological niche of a species as the set of physical and biological conditions under which the species can maintain its population without immigration. For a species to maintain its population, individuals must be able to survive and reproduce, obtaining energy and nutrients while avoiding predation. Hutchinson (1959) further defined the ecological niche concept by distinguishing between the fundamental and realized niches. The fundamental niche consists of the total potential area that meets all the physical and biological requirements of a species; the actual distribution of a species is determined by a variety of factors such as dispersal, history, and physical barriers—an area described as the realized niche. This distinction is particularly important in the ENM of *C. gattii* because the present observed distribution of *C. gattii* in BC (i.e., its realized niche) may not yet reflect its full potential geographic range in BC (i.e., its fundamental niche).

A common challenge in determining the geographic distribution and range of plant and animal species is the lack of data, especially in remote areas. Extensive sampling is typically required, but in most cases, field sampling is prohibitively expensive and limited by terrain. Absence data are even more scarce because it is difficult to determine when a species does not (transiently) exist in an area (Guisan and Thuiller 2005). However, when species observation data are available, ENM can provide accurate forecasts of a species’ ecological niche based on the environmental characteristics of known species locations.

*C. gattii* is an invasive species, one that is expanding into ecological niches that it has not previously been known to occupy. As such, information derived from its distribution in previously known habitats cannot be used to inform the development of our ENM (contra Guisan and Thuiller 2005). Furthermore, given that we must assume that it has not yet achieved environmental equilibrium, using methods that rely on (pseudo) absence data, such as generalized linear models or generalized additive models, is problematic (Guisan and Thuiller 2005).

## The GARP methodology

We selected the Genetic Algorithm for Rule-set Prediction (GARP) software package for our study because it has a proven record of accuracy and performance for predicting the ecological niches of a variety of species (Peterson 2001; Soberon and Peterson 2005; Stockwell and Peterson 2002).

GARP, developed by Stockwell and Peters (1999), is an iterative, artificial-intelligence–based approach to ENM (Peterson 2001). It employs a “superset” of rules to identify the ecological niche of a species based on nonrandom correlations among species presence, species absence, and environmental parameter values (University of Kansas Center for Research 2002). GARP divides the species occurrence data (model input points) into training and testing data sets, and then environmental data layers relevant to the ecology of the species in question are added to construct the model. Typical environmental data sets used for ENM include topographic (elevation, aspect, slope), climatic (temperature, precipitation), and biological (vegetation, other species distributions) data layers, a much broader array of predictive variables than that used in climate envelope models (Stockwell and Peters 1999).

GARP employs four types of rules to identify the ecological niche of a species: atomic, range, negated range, and logistic regression (Payne and Stockwell 1996). The rules for predicting ecological niche of a species are randomly developed and progressively employed on the training data set. At each iteration, the predictive accuracy of the model is evaluated based on the testing data set (Peterson et al. 2002). The rule is accepted and incorporated into the model if the change in predictive accuracy increases; otherwise, the rule is rejected and dropped. A one-tailed chi-square test on the difference between the probability of the predicted value before and after the employment of the rule is used to determine the statistical significance of these rules (Stockwell and Peters 1999). The model runs 1,000 iterations or until convergence is reached.

Predictive accuracy of the model—defined as the sum of all correct predictions (both presence and absence) divided by the sum of all predictions—is a function of the number of correct predictions and of errors of commission and omission (University of Kansas Center for Research 2002). A commission error occurs when the model predicts a species to occur where it does not (i.e., including areas not actually inhabited); an omission error occurs when the model fails to predict a species occurrence where it does in fact occur (i.e., excluding areas actually inhabited).

## Materials and Methods

### Geographic information systems and modeling software

We used ArcGIS 9.1 and ArcView 3.2 (Environmental Systems Research Institute, Redlands, CA, USA) to perform geographic information system (GIS) mapping. The Geostatistical and Spatial Analyst extensions were used to interpolate continuous surface layers and manipulate raster data sets for ENM in Desktop GARP 1.1.6 (University of Kansas Biodiversity Research Center 2002).

### Disease surveillance data

**Figure 1:**
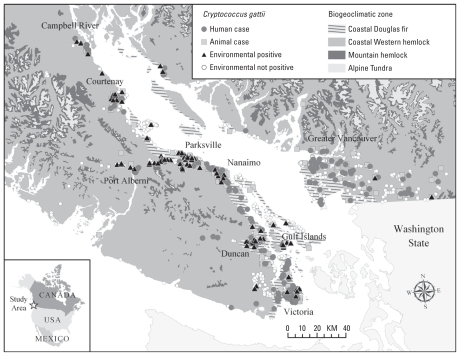
Geographic distribution of human and animal cryptococcosis and isolation of *C. gattii* from the environment in southwestern BC. Cases are mapped by address of residence and include travel-related cases to Vancouver Island. Figure adapted from Kidd et al. (2007b), by permission from the American Society for Microbiology.

We used *C. gattii* surveillance and environmental sampling data as input data to build and test the ecological niche models (Figure 1). Human cases were reported to public health officials by family physicians, microbiologists, pathologists, and respirologists. Shortly after the initial identification of cryptococcosis due to *C. gattii* in BC, the BC Centre for Disease Control developed an enhanced disease surveillance program to identify new cases of cryptococcosis. A formal case definition, detailed questionnaire, and standardized diagnostic laboratory tests were created. Public health officials interviewed each case to collect demographic information, address of residence, travel history, medical history, and environmental exposure. In 2003, cryptococcosis due to *C. gattii* became a reportable disease in BC.

Animal cases were detected and reported by veterinarians, and with the animal owner’s consent this information was provided to public health officials. In particular, Duncan et al. (2005) performed an extensive review of medical records from sentinel veterinary clinics on Vancouver Island and BC mainland and developed an animal cryptococcosis database. Subsequent animal cases have been recorded and managed by K. Bartlett and S. Lester (unpublished data). Geocoding of animal and human cases by street address or postal code of residence was performed using ArcGIS. The use of human and animal case data in the present study (i.e., geographic location of each case for the modeling) was reviewed and approved by the University of British Columbia Behavioral Research Ethics Board (study number H07-01235).

Environmental sampling for *C. gattii* was conducted on trees, soil, air, and water (for detailed methods, including limits of detection, see Kidd et al. 2007b; University of British Columbia *Cryptococcus* Research 2006). Sampling was conducted in areas with and without associated cases of *C. gattii* cryptococcosis, although most samples were taken in neighborhoods with human or animal cases, including properties where the cases resided, and public trails and recreational parks (Kidd et al. 2007b). Longitudinal sampling in select locations was performed to determine whether a seasonal pattern of *C. gattii* presence in the environment existed (i.e., whether a location was “permanently” positive or “transiently” positive for *C. gattii*). A recreational grade (~ 15 m spatial accuracy) Global Positioning System receiver (eTrex, Garmin, Olathe, KS, USA) was used to record the latitude and longitude coordinates from where the samples were collected, which was then mapped using ArcGIS.

### Environmental data layers

We used 15 environmental data layers believed to be relevant to *C. gattii* biogeography in the ENM: elevation, aspect, and slope (all elevation-related data were derived from GTOPO30; U.S. Geological Survey 1996); biogeoclimatic zone (BC Ministry of Forests and Range 2006); January and July average, maximum, and minimum temperature and annual, January, and July total precipitation (all climate-related data were derived from 1971–2000 “normals”; Environment Canada 2001; National Oceanic and Atmospheric Administration 2006); and soil drainage and development (Agriculture and Agri-Food Canada 1995). Each environmental data layer had complete geographic coverage across the province. Based on the spatial resolution of the GTOPO30 digital elevation model, the cell size of the raster data layers was 600 m × 600 m. A total of 2,628,833 cells equating to 946,380 km^2^ spatial coverage were used in the ENM of *C. gattii* in BC. These data are described in more detail in the Supplemental Material (doi:10.1289/ehp.0901448) and in Mak (2007).

### GARP modeling of C. gattii

Locations of *C. gattii* human and animal cases and environmental positive samples were used, in separate models, to train and test the respective models. GARP is able to use a variety of different data types in the ENM process because it employs a number of different rule types (atomic, range, negated range, and logistic regression). The *C. gattii*, aspect direction, biogeoclimatic zone, and soil data sets were nominal data. The elevation, slope, temperature, and precipitation data sets were ratio data.

#### *C. gattii* data used in the ENM

Human and animal cases within the known endemic areas for *C. gattii* [i.e., along the south-central east coast of Vancouver Island from Campbell River (N50°01′ W125°14′) in the north to Greater Victoria (N48°26′ W123°22′) in the south, and the Gulf Islands (N48°57′ W123°32′)] were used as the ENM input data points. These locations, mapped by address of residence, do not definitively represent the presence of *C. gattii*in the local area or where exposure to *C. gattii* occurred, but instead represent proxies for where exposure may have likely occurred because people, and especially companion animals, tend to spend most of their time and activities around their area of residence. Travel-related cases from the BC mainland and from areas currently nonendemic on northern Vancouver Island were excluded from the ENM process. In all, 122 human and 135 animal cases from endemic areas on Vancouver Island and the Gulf Islands were used in the ENM of *C. gattii*.

We also used (384) positive environmental sampling locations from permanently established sites as ENM input data points. These locations represent confirmed sites of *C. gattii* presence.

#### Data used for model validation

A small number of mainland cases (11 human and 13 animal), with precise location information and without travel to Vancouver Island or other endemic sites, were identified on the BC mainland. Fifty-five transiently positive environmental sampling locations, including seven samples from four sites from the BC mainland, were also identified. The locations of these mainland cases and transient positive environmental samples were used to verify the predictive accuracy of the *C. gattii* ENM because the empirical data from Vancouver Island were used to forecast the ecological niche of *C. gattii* on the BC mainland.

#### Environmental data layer jackknifing

Desktop GARP can identify which environmental factors are more important than others for predicting the ecological niche of a species by “jackknifing” environmental data layers, which involves running multiple ENM tasks with only a subset of the layers (Stockwell and Peters 1999). The jackknifing procedure determines which environmental data layers contribute positively to the model’s performance; a good model has small commission and omission errors and high training and testing accuracy.

The human and animal cases and positive environmental sampling locations were divided into training (70%) and testing (30%) sub-data sets. All 15 environmental data layers were jackknifed individually through 30 model runs, for a total of 450 model runs for each scenario. Each model run consisted of a maximum of 1,000 iterations or until the convergence limit of 0.01 was reached.

#### GARP modeling with significant environmental data layers only

A final ENM of *C. gattii* was performed using only the significant environmental data layers identified in the jackknifing procedure. For these final runs, the input *C. gattii* data sets were again divided into training (70%) and testing (30%) sub-data sets, and 20 model runs were performed. A separate binary predictive layer was created from each model run with training and testing accuracy > 95%; cell values of 1 indicate locations with appropriate ecological conditions to support *C. gattii*, whereas values of 0 indicate unsuitable locations. “Optimal” *C. gattii* ecological niche areas had 11–20 model output grid data layer agreement, and “potential” *C. gattii* ecological niche areas had 1–10 model output grid data layer agreement.

## Results

### Environmental data layer jackknifing

The elevation, biogeoclimatic zone, and January average, maximum, and minimum temperature data layers had training and testing accuracy > 95% (*p* < 0.001) and small commission and omission errors, based on jackknifing the environmental data layers with the distribution of *C. gattii* cases and positive environmental sampling locations as the dependent variables. July minimum temperature and July total precipitation also had training and testing accuracy > 95% (*p* < 0.001) based on the jackknifing of environmental data layers with the distribution of animal cases.

Conversely, across all scenarios, aspect, slope, July maximum temperature, annual total precipitation, soil drainage, and soil development were poor predictor variables for modeling the ecological niche of *C. gattii* in BC because their commission and omission errors were large and because their training and testing accuracy were low. The complete set of results is available in Supplemental Material (doi:10.1289/ehp.0901448) and Mak (2007).

### Ecological niche model predictions

#### Ecological niche model prediction maps

**Figure 2:**
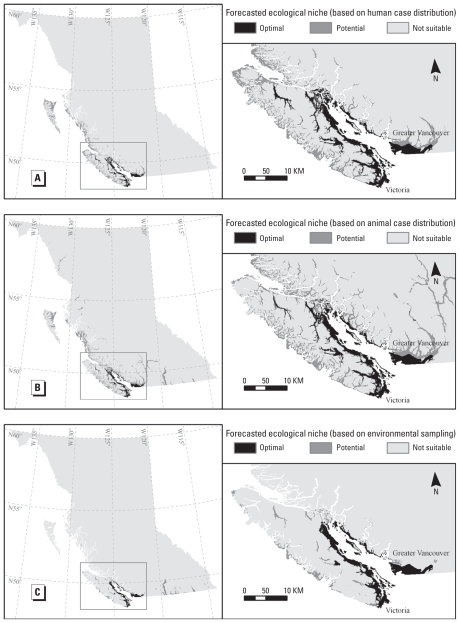
*C. gattii* ecological niche prediction maps based on the distribution of human (*A*) and animal (*B*) cases and positive environmental sampling locations from permanently established sites (*C*) in BC.

We created *C. gattii* ecological niche prediction maps for each ENM scenario: human cases, animal cases, and positive environmental sampling locations (Figure 2), using only significant environmental data layers (Table 1). Geographic similarities among the *C. gattii* ecological niche prediction maps were the presence of optimal ecological niches along the central and south eastern coast of Vancouver Island, Gulf Islands, Sunshine Coast (N50°00′ W123°45′), and Vancouver Lower Mainland (N49°15′ W123°00′). The *C. gattii* ecological niche predictions based on the environmental sampling locations produced the most conservative results. For example, potential *C. gattii* ecological niche areas on the Queen Charlotte Islands (N53°00′ W132°00′) and central BC coast are reduced or absent in the positive environmental sampling locations results (Figure 2C), whereas the *C. gattii* ENM scenarios based on the distribution of human and animal cases identify these as potential ecological niche areas (Figure 2A, B). We believe that the conservative model prediction based on environmental sampling data is a result of the stringent inclusion of *C. gattii*–positive data from only established locales, and the more liberal model predictions based on case data are a result of the spatial uncertainty related to using address of residence as a proxy for where *C. gattii* exposure occurred.

#### Validation of the ecological niche model predictions

We used the nontravel (i.e., locally acquired) human and animal *C. gattii* cases and positive environmental samples on the BC mainland to independently validate the ecological niche model predictions, because we based the models on Vancouver Island and Gulf Islands *C. gattii* observations (current endemic areas for *C. gattii* in BC). We overlaid the 11 human and 13 animal *C. gattii* cases and 4 positive environmental sampling sites on the BC mainland with the forecasted *C. gattii* ecological niche models.

All 11 human cases, 9 of 13 animal cases, and 3 of 4 positive environmental sampling sites on the BC mainland were located in the forecasted *C. gattii* ecological niche areas based on the ENM scenario using the distribution of human *C. gattii* cases to construct the model. The remaining four animal cases and one positive environmental sampling site were located within 2.5 km of the nearest forecasted *C. gattii* ecological niche areas.

**Figure 3:**
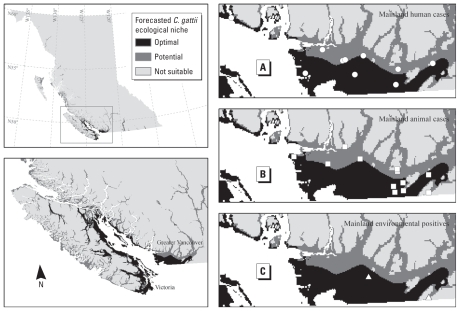
Validation of *C. gattii* ENM predictions on the BC mainland based on the model input of animal cases. The distribution of mainland human (*A*) and animal (*B*) cases and positive environmental samples (*C*) are displayed in the insets. Not shown are 1 of 11 human cases, 1 of 13 animal cases, and 2 of 4 positive environmental sampling sites, which are located in the northern Sunshine Coast area, outside of the inset map’s extent.

Using the distribution of animal *C. gattii* cases to construct the model, all 11 human cases, all 13 animal cases, and all 4 positive environmental sampling sites on the BC mainland were located in the forecasted *C. gattii* ecological niche areas (Figure 3).

Finally, using the distribution of positive *C. gattii* environmental sampling locations to construct the model, 6 of 11 human cases, 6 of 13 animal cases, and 3 of 4 positive environmental sampling sites on the BC mainland were located in the forecasted *C. gattii* ecological niche areas. The remaining five human cases, seven animal cases, and one positive environmental sampling site were located within 3.0 km of the nearest forecasted *C. gattii* ecological niche areas.

Based on these results, the use of animal case data to construct the ecological niche model of *C. gattii* produced the best modeling results and highest predictive power for *C. gattii* presence on the BC mainland.

#### *C. gattii* ecological niche characterization

Table 2 describes the environmental characteristics of the forecasted ecological niche of *C. gattii* based on ENM using environmental data layers with > 95% accuracy to construct the models. Low-lying elevations (< 770 m and averaging 100 m above sea level), daily January average temperatures > 0°C, and presence within the coastal Douglas fir and the very dry regions of the coastal western hemlock biogeoclimatic zones are strongly associated with the ecological niche of *C. gattii*. Of particular note is the association of daily January average temperatures > 0°C within the forecasted ecological niche areas of *C. gattii*, and the general absence (or transiently positive nature) of *C. gattii* in geographic areas where the daily January average temperatures are < 0°C. This finding suggests that *C. gattii* may not colonize regions with prolonged, below-freezing temperatures.

The ENM of *C. gattii* based on the distribution of animal cases to construct the model also identifies mild daily July minimum temperatures (11.5°C) and moderate July precipitation (41 mm) in areas associated with *C. gattii* in the environment.

## Discussion

### Appropriateness of the model properties, methods, and data

The validity of any ecological niche model is dependent on the modeling methods, quality, and completeness of the data on species occurrence and on the quality and completeness of the predictor environmental data layers used in the model (Ron 2005). Therefore, it is important to note that the cryptococcosis surveillance data used in this study do not definitively represent where *C. gattii* is found in the environment. We mapped cases by their address of residence, so these case locations were proxies to where exposure to *C. gattii* may have occurred. Unfortunately, from an ENM and epidemiologic perspective, the high mobility of people in developed countries such as Canada, and the duration of time between exposure to *C. gattii* and onset of symptoms (typically between 3 and 9 months), makes identifying the actual place of exposure difficult to ascertain. Nonetheless, ENM of *C. gattii* based on the distribution of human cases is appropriate, but caveats pertaining to the limitations of the data, and possible resulting model errors due to the “uncertainty” of these data (whether each case truly represents the presence of *C. gattii* in the area), should be noted and understood.

Animal cryptococcosis surveillance data, especially the feline cases, may be more appropriate for identifying the true presence of *C. gattii* in an area because companion animals travel less and because their contact with soil and woody material (environs where *C. gattii* are most likely to be encountered) are more direct than those to humans—thereby providing more reliable location data for the ENM of *C. gattii* than human case data. Currently, the number of reported animal cases is approximately 1.5 times greater than the number of reported human cases. New reports of cryptococcosis in previously nonendemic *C. gattii* geographic areas in BC have been identified first in animal cases and then in human cases. However, although a greater number of animal records have been reported to veterinary and public health, the detail of disease surveillance and other relevant information relating to travel history is generally less complete than those of the human cases. Also, a rigorous system for reporting and tracking animal *C. gattii* cases is not in place at the present time.

Preselection bias in the selection of environmental locations sampled near identified human and animal cases and in environs frequented by humans may have introduced modeling errors. However, the data used included some random sampling for *C. gattii*, which was performed during the initial stages of the environmental sampling strategy before *C. gattii* had been isolated from the BC environment. For example, random samples were obtained from the west coast of Vancouver Island, Gulf Islands, Sunshine Coast, and Vancouver Lower Mainland (Figure 1). These samples were part of the overall environmental sampling data set we used in the ENM of *C. gattii* in BC.

Our ENM of *C. gattii* used only the permanently positive environmental sampling locations (following Guisan and Thuiller 2005); we did not include the transiently positive environmental sampling locations as input points for the ecological niche model because evidence of true colonization of *C. gattii* in the environment had not been established. Spatial overlay of the forecasted *C. gattii* ecological niche models and transiently positive locations showed that 47 of 55 transiently positive locations were located within the forecasted optimal *C. gattii* ecological niche based on the distribution of human cases, 44 were located within the forecasted optimal *C. gattii* ecological niche based on the distribution of animal cases, and 41 were located within the forecasted optimal *C. gattii* ecological niche based on the distribution of permanently positive environmental sampling locations.

#### Interpretation of the ecological niche model prediction maps

Only a small percentage of the province (< 2%) is geographically located within the forecasted ecological niche area of *C. gattii* (Figure 2). However, approximately two-thirds of BC’s population resides in this area, primarily in the Vancouver Lower Mainland and central to southern east coast of Vancouver Island. A small number of positive environmental samples have been isolated on the BC mainland in the past 2 years, but subsequent, repeat environmental sampling has not isolated it in the same locations. Furthermore, the incidence and rate of human illness on the BC mainland are still disproportionately low compared with the rates observed on Vancouver Island. The ENM of *C. gattii* performed in this study identifies, however, that the Vancouver Lower Mainland has optimal ecological niches that can support the establishment of *C. gattii* in the local environment. If *C. gattii* does become established in the Vancouver Lower Mainland in concentrations similar to the endemic areas on Vancouver Island, and if similar rates of infection experienced in the endemic areas on Vancouver Island are also experienced in the Vancouver Lower Mainland, we would expect to see 79 cases of cryptococcosis per year (36 cases per million population on Vancouver Island per year during 2002–2005 multiplied by 2.2 million population in the Vancouver Lower Mainland) in the Vancouver Lower Mainland alone. Finally, based on the characteristics of optimal ecological niche areas for *C. gattii* identified in this study, we hypothesize that the San Juan Islands and Puget Trough of Washington State and the Willamette Valley of Oregon State will also eventually become endemic areas for *C. gattii* as anthropogenic, wind, and mechanical dispersal of *C. gattii* into these areas continue.

## Conclusion

In summary, our study found that the “optimal” ecological niche areas of *C. gattii* in BC are limited to the central and south eastern coast of Vancouver Island, the Gulf Islands, Sunshine Coast, and Vancouver Lower Mainland. Small, isolated geographical areas on the Queen Charlotte Islands, the BC central coast, west coast of Vancouver Island, and southern BC interior have environmental conditions that could potentially support the establishment of *C. gattii*. These areas are characterized by low-lying elevations, daily January average temperatures > 0°C, and presence within the coastal Douglas fir and very dry regions of the coastal western hemlock biogeoclimatic zones. In particular, freezing temperatures experienced in areas throughout the rest of BC appear to be the primary environmental limiting factor for *C. gattii*.

The predictive accuracy of the *C. gattii* ecologic niche models was extremely high: training and testing accuracy was > 98%, and fewer than two commission and omission errors were committed per model run. The use of human and animal cryptococcosis surveillance data to model the ecological niche of *C. gattii* was effective and corresponded well to the forecasted ecological niche of *C. gattii* based on the distribution of positive environmental sampling locations. In particular, animal surveillance data proved to be good indicators for *C. gattii* presence in an area. This finding suggests that surveillance for animal cryptococcosis may be useful as an early alert system for human disease in BC. Therefore, we recommend the creation of a comprehensive, provincewide animal cryptococcosis surveillance system to track laboratory confirmed and clinically diagnosed cases of cryptococcosis due to *C. gattii*.

## Figures and Tables

**Table 1:** Summary of *C. gattii* ENM results based on human and animal cases and on positive environmental sampling locations from permanently established sites.

	Errors		Accuracy (%)	
*C. gattii* ENM scenario	Commission	Omission	Training	Testing
Human (five layers)	1.2	0.4	99.4	99.2
Animal (seven layers)	1.2	0.5	98.7	98.3
Environmental (five layers)	0.7	0.0	99.7	99.7

*p*-Values in all cases were < 0.001.

**Table 2:** Summary of environmental characteristics of the forecasted optimal *C. gattii* ecologic niche in BC based on the distribution of human and animal cases and permanently established *C. gattii* sites.

	Human			Animal			Environmental		
Data layer	Avg	Max	Min	Avg	Max	Min	Avg	Max	Min
Elevation (m)	120	762	1	106	762	1	95.0	448	1
January									
Avg temp^a^	2.8	4.7	0.9	2.9	5.6	1.1	2.9	5.6	0.7
Max temp	5.4	7.8	3.3	5.5	7.8	3.5	5.6	7.8	3.2
Min temp	0.1	3.9	−2.0	0.2	3.9	−2.0	0.1	3.9	−2.2
July									
Min temp	—	—	—	11.5	15.2	8.1	—	—	—
Precip (mm)	—	—	—	41.0	111.3	15.5	—	—	—
Biogeoclimatic zone	Area (km^2^)	Area (%)		Area (km^2^)		Area (%)	Area (km^2^)		Area (%)
	
Coastal Douglas fir	2,383			2,378		28.5	2,385		37.0
Coastal Western hemlock (very dry)	6,353	70.8		5,713		68.5	4,053		63.0
All other zones	243	2.7		251		3.0	—		—

Abbreviations: Avg, average; max, maximum; min, minimum; precip, precipitation; temp, temperature (°C).
